# Bio-inspired dewetted surfaces based on SiC/Si interlocked structures for enhanced-underwater stability and regenerative-drag reduction capability

**DOI:** 10.1038/srep24653

**Published:** 2016-04-20

**Authors:** By Junghan Lee, Zhuo Zhang, Seunghyun Baek, Sangkuk Kim, Donghyung Kim, Kijung Yong

**Affiliations:** 1 Surface Chemistry Laboratory of Electronic Materials, Department of Chemical Engineering, POSTECH (Pohang University of Science and Technology), Pohang, 790-784 Korea

## Abstract

Drag reduction has become a serious issue in recent years in terms of energy conservation and environmental protection. Among diverse approaches for drag reduction, superhydrophobic surfaces have been mainly researched due to their high drag reducing efficiency. However, due to limited lifetime of plastron (i.e., air pockets) on superhydrophobic surfaces in underwater, the instability of dewetted surfaces has been a sticking point for practical applications. This work presents a breakthrough in improving the underwater stability of superhydrophobic surfaces by optimizing nanoscale surface structures using SiC/Si interlocked structures. These structures have an unequaled stability of underwater superhydrophobicity and enhance drag reduction capabilities,with a lifetime of plastron over 18 days and maximum velocity reduction ratio of 56%. Furthermore, through photoelectrochemical water splitting on a hierarchical SiC/Si nanostructure surface, the limited lifetime problem of air pockets was overcome by refilling the escaping gas layer, which also provides continuous drag reduction effects.

Dewetted surfaces have attracted a lot of attention because of their wide range of potential applications, such as for anti-fouling surfaces^1^,^2^,^3^,^4^,^5^,^6^,^7^, waterproof devices^8^,^9^,^10^, microchannels^11^,^12^, anti-icing^13^,^14^,^15^,^16^,^17^, oil/water separation^18^,^19^,^20^, drag reduction^21^,^22^,^23^ and other non-wetting related fields^24^,^25^,^26^,^27^,^28^,^29^,^30^,^31^,^32^. Among those application fields of dewetted surfaces, drag reduction is one of the most significant issues for energy conservation and environmental protection, which have become global concerns in the last few decades. Especially in the fields of marine vessels and fluid channels, drag reduction can tremendously reduce consumption of energy and resources^33^,^34^. Various surface structures and morphologies have been researched to induce drag reduction effects, including compliant coatings^35^,^36^, polymer coatings^37^,^38^,^39^, surfactants^40^,^41^,^42^, microbubbles^43^,^44^, and superhydrophobic coatings^23^,^45^. Among the various strategies for drag reduction, superhydrophobic surfaces, which are dewetted surfaces that mimic lotus leaves, have shown dominant efficiency in drag reduction. However, the instable underwater superhydrophobicity has impeded their use in practical applications^46^,^47^,^48^.

In superhydrophobic surfaces, it is known that the presence of an air (or gas) interlayer on the submerged surface causesthe non-wetting behavior, and thus, the stability of underwater superhydrophobicity is determined by the lifetime of the air interlayer^49^. However, the air interlayer (plastron) is highly unstable with limited lifetime due to the diffusion of the air gases into water^50^. According to previous researches, the gas diffusion rates are mainly determined by surface characteristics (surface morphology and surface energy) and hydrostatic pressure^51^,^52^. Various surface structures such as mesoporous structures, nanowire arrays, and micro/nano hierarchical structures, have been reported to enhance the lifetime of the air interlayer^53^,^54^. However, in spite of those diverse studies, they could not overcome the impermanence of the air interlayer.

In this study we have developed new SiC/Si interlocked hierarchical structures using a carbothermal reduction based synthesis method^55^. Compared to previously reported structures, our surface drastically improved the lifetime of the air interlayer and showed the highest stability of underwater superhydrophobicity due to its unique networking structure^47^,^56^. According to our drag reduction measurements, our superhydrophobic SiC/Si hierarchical surface showed 56% drag reduction effect compared to a flat Si surface. Conversely, the superhydrophilic SiC/Si surface exhibited a drag enhancement property.

Additionally, to solve the limited lifetime problems of air interlayers in underwater, a photoelectrochemical (PEC) reaction, which generates hydrogen gas through solar water splitting, was employed in our system to refill the escaping air interlayers^57^,^58^. The underwater superhydrophobicity was successfully restored by the PEC reaction of the SiC/Si interlocked net structures and provided a regenerative drag reduction capability as a results. Our study presents a novel hierarchical interlocked net structure with a high gas capture capability and also presents an unprecedented method for providing a continuously regenerating drag reduction property.

## Results

### Fabrication of SiC nanowire arrays and SiC/Si hierarchical structures

**Figure 1:**
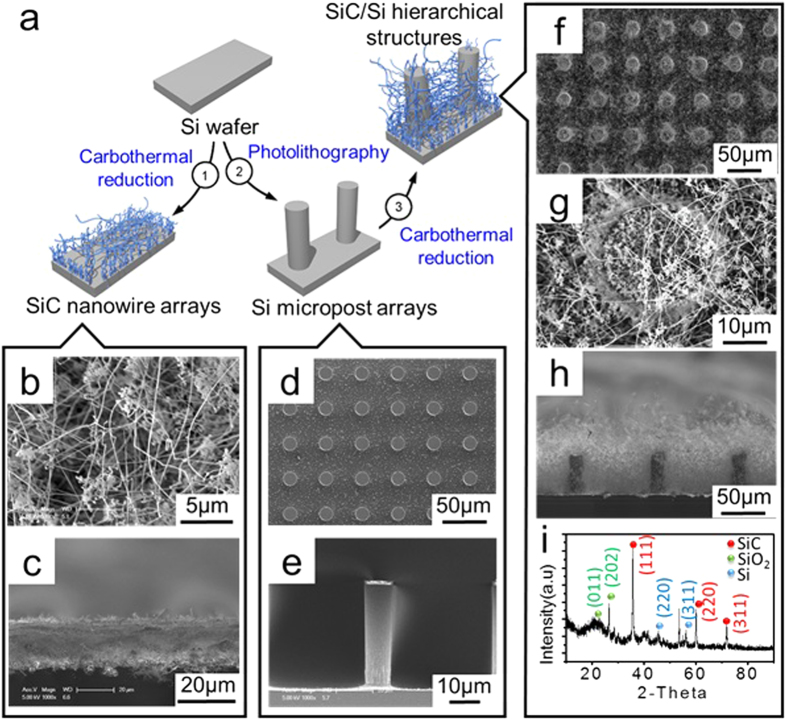
(**a**) Scheme for the fabrication process of a SiC/Si hierarchical structure. (**b,c**) Top and cross-sectional SEM images of SiC nanowire arrays. (**d,e**) Top and cross-sectional SEM images of Si micropost arrays. (**f–h**) Top, magnified and cross-sectional SEM images of SiC/Si hierarchical structures. (**i**) XRD patterns of as-prepared SiC/Si hierarchical structures.

A SiC nanowires arraywith high density was synthesized by carbothermal reduction of WO_3_ and graphite on a NiO-catalyzed p-type Si substrate^55^,^59^. As shown in Fig. 1b, the SiC nanowire arrays had rough and randomly aligned interlocked net structures with a high aspect ratio of 20–50 nm diameter and were tens of micrometers in length. Also the SiC nanowires were stacked layer upon layer with a uniform thickness of ~20 μm (Fig. 1c). The growth of highly dense, long and stacked SiC nanowires was based on solid-liquid-solid (SLS) mechanism, in which the Si substrate is used as a Si source material for SiC nanowires formation. Carbothermal reduction of WO_3_ and graphite produces CO_x_ gases, which are used as a carbon source for the growth of SiC. Then, the reaction between SiO_x_ and CO_x_ produces SiC, which nucleates on the surface and grows into SiC nanowires.

For the growth of the hierarchical SiC/Si structures, Si micropost arrays were simultaneously used asa Si source and substrate. The growth procedure is illustrated in Fig. 1a.The Si micropost arrays were fabricated by a typical photolithography method. The Si microposts had a height of ~50 μm, diameter of ~20 μm, and the pitch between the micropostswas ~30 μm (Fig. 1d,e). Si microposts with various pitches (50 and 100 μm) were also prepared using the appropriate Cr photomasks (Supplementary Fig. 1). After heating the NiO-catalyzed Si micropost samples at 1100 °C for 3 hours with the help of WO_3_/C powder carbothermal reduction, a highly dense and stacked growth of SiC nanowires was observed on the Si microposts showing a similar morphology as that of SiC nanowire arrays grown on the Si substrate except for the existence of regularly spaced Si microposts (Fig. 1f,g). Because the top and side faces of the Si microposts acted as growth sites for SiC, far denser SiC nanowires were found on the Si microposts compared to inter-spaces between Si microposts. Additionally, the SiC/Si hierarchical structures had a uniform thickness of ~80 μm (similar to the height of the Si micropost arrays) without any micrometer scale roughness(Fig. 1h). The XRD pattern of the SiC/Si hierarchical structures is shown in Fig. 1i. It shows the typical XRD peaks of the β-SiC crystal, Si microposts that correspond to SiC/Si hierarchical structures. The XRD peak of SiO_2_was also observed due the oxidation of SiC and the Si microposts during the thermal heating reaction of the SiC nanowires growth.

### Wettability control of SiC nanowire arrays and SiC/Si hierarchical structures

**Figure 2:**
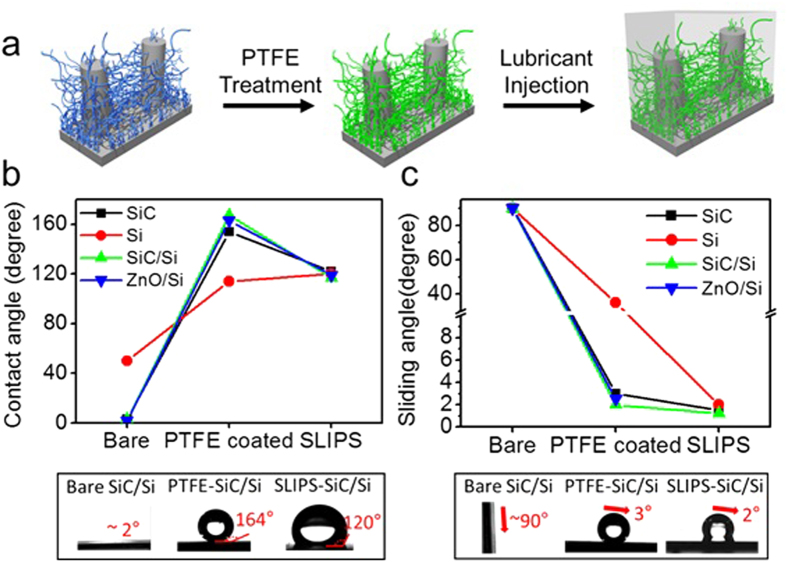
(**a**) Schematic procedures for fabricating superhydrophobic and slippery liquid infused SiC/Si hierarchical structures. (**b**) Static water contact angle (CA) transitions of bare, PTFE- coated (superhydrophobic) and lubricant injected (slippery liquid infused) SiC nanowire arrays, Si micropost arrays, SiC/Si hierarchical structures and ZnO/Si hierarchical structures. (**c**) Water sliding angle (SA) transitions of bare, PTFE-coated (superhydrophobic) and lubricant injected (slippery liquid infused) SiC nanowire arrays, Si micropost arrays, SiC/Si hierarchical structures and ZnO/Si hierarchical structures.

The surface wettability control process of as-synthesized surfaces is depicted in Fig. 2a. Theas-synthesized bare SiC nanowire arrays and SiC/Si hierarchical structures both showed superhydrophilic properties with static water contact angles (CAs) below 5°and water sliding angles (SAs) of ~90° (Fig. 2b,c). This hydrophilicity is due to the hydrophilic -OH groups of the SiO_2_ layers that formed on the SiC surfaces during the thermal reaction process. The bare Si micropost samples showed a moderate hydrophilic wettability with water CAs of ~50° and water SAs of ~90°. To reduce the surface energy and make the surfaces hydrophobic, a polytetrafluoroethylene (PTFE) solution was spin coated on the sample surfaces. Due to the C-F chains of PTFE, the self-assembled-monolayer (SAM) layer of PTFE coatedon the surfaces exhibited a high water repellency. The water CAs ofthe SAM-modified SiC nanowire arrays and SiC/Si hierarchical structures were 154° and 164°, respectively, which shows their superhydrophobicity. The SiC/Si hierarchical structure was extremely superhydrophobic due to the enhanced surface roughness. Also both sample surfaces had water SAs below ~3° showing their very low adhesion affinity with water droplets. The PTFE-coated Si micropost arrays showeda moderate hydrophobicity with water CAs of 114° and water SAs of 35° due to the lack of the surface roughness. As a reference of comparison with other hierarchical nanostructure, PTFE-coated ZnO/Si hierarchical structures were prepared. In the case of theZnO/Si hierarchical structure, rather short ZnO nanorods grew on the Si microposts; the experimental details and SEM images are presented in the Supplementary Fig. 2. These PTFE-coated ZnO/Si hierarchical structures also showed superhydrophobicity with water CAs of 163° and water SAs of 2.5°.

Because slippery liquid infused surfaces(SLIPSs) have recently attracted attention as drag reducing surfaces^60^,^61^,^62^,^63^,^64^, SLIPS samples were also prepared for a comparison study by infusing a perfluoropolyether lubricant (Krytox from Dupont) into the SiC nanowire arrays, Si micropost arrays, SiC/Si hierarchical structures and ZnO/Si hierarchical structures, respectively. The four as-fabricated SLIPSs showed similar water CAs of ~120° and water SAs of ~2°, regardless of their solid surface structures. The reason for these low SAs was that the solid surfaces did not directly contact the water droplets in the SLIPS because the lubricant fully infiltrated the micro/nano structures and made very smooth, homogeneous overlayers. The lack of pinning and adhesion of water droplets due to the lubricant overlayers led to a high water repellency with a low water contact angle hysteresis and water SAs as shown in Fig. 2.

### Stability analysis of superhydrophobic SiC nanowire arrays and SiC/Si hierarchical structures in underwater conditions

**Figure 3:**
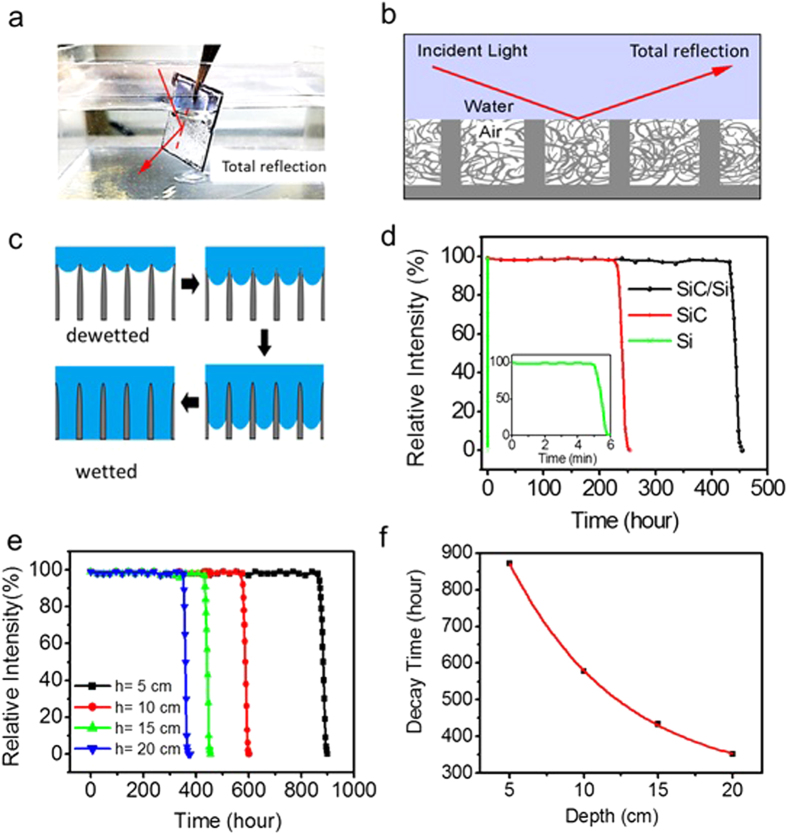
(**a**) Digital image of superhydrophobicSiC/Si hierarchical structures submerged in water. (**b**) Schematic images for total reflections at the water-air interface of SiC/Si hierarchical structures. (**c**) Schematic images for the diffusion process of the air interlayer in water. (**d**) Relative intensity transitions and τ_d_ values of SiC nanowire arrays, Si micropost arrays and SiC/Si hierarchical structures when submerged in water at the depth of 15 cm. (**e**) Relative intensity transitions of SiC/Si hierarchical structures at 4 different immersion depths (5, 10, 15 and 20 cm). (**f**) Graph of τ_d_*vs* the immersion depth of the submerged surfaces.

The as-prepared superhydrophobic SiC nanowire arrays and SiC/Si hierarchical structures showed a visual change when they were submerged in water (Fig. 3a). When the incident angle of light is greater than a critical angle (θ_c_ = 48° for the water/air interface) of total reflectance, the air interlayer reflects all the incident light and submerged surfaces look mirror-like and silvery (Fig. 3b).

The effects of the surface structures on the stability of the underwater superhydrophobicity were investigated by measuring the lifetime of the air interlayer for three different structures: SiC nanowire arrays, Si micropost arrays and SiC/Si hierarchical structures. The photo-image of the submerged superhydrophobic surface initially consisted of white pixels due to the mirror-like total reflection but the pixels gradually turned dark when the gas in the air interlayer began to diffuse into the water. The diffusion process of the air interlayer is depicted in Fig. 3c. Plotting the ratio of white pixels to dark pixels allowed the superhydrophobicity lifetimes to be quantified. The relative intensity (defined as the ratio of white pixels to total pixels) was plotted as the function of immersion time of the samples. The immersion depth of the samples was fixed to 15 cm for the underwater stability tests. As shown in Fig. 3d, regardless of the surface structures, the underwater superhydrophobicity was preserved for a certain time, but degraded in a very short time. To analyze the decay of the underwater superhydrophobicity quantitatively, the decay time (τ_d_), which was defined as the time at which the ratio of white pixels to dark pixels reached 90%, was measured. The superhydrophobic Si micropost arrays with a 30 μm pitch had a τ_d_ of 5 min and completely lost their superhydrophobicity in 10 min. In contrast, the SiC nanowire arrays had a τ_d_ of ~230 hr. This enormous difference in τ_d_ is due to the Laplace pressure (p_L_) induced by the capillary force^46^,


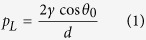


where γ is the water surface tension, *θ*_*o*_ is the water CAs on the flat surface and *d* is the distances between adjacent micro or nano structures. The Laplace pressure is the force that inhibits water intrusion into the structure. As SiC nanowire arrays had much smaller spacings between adjacent nanowires (a few nanometers) compared to those in the Si micropost arrays, the SiC nanowire arrays had a much larger Laplace pressure and a highly stable underwater superhydrophobicity. The superhydrophobic SiC/Si hierarchical structures showed a τ_d_ of ~434 hr (over 18 days), about two times longer than that of the SiC nanowire arrays. This enhanced stability resulted from the taller stack height of the SiC/Si hierarchical structures(50 μm) than that in the SiC nanowire arrays (20 μm). Thus the intrusion of water into the micro/nano structures of SiC/Si took longer time than with the SiC nanowire arrays, even though both had similar Laplace pressures.

The underwater superhydrophobicity stability of the SiC/Si hierarchical structures was also measured at different immersion depths to examine the effect of hydrostatic pressure on the stability of underwater superhydrophobicity. As the immersion depth was increased to 5, 10, 15 and 20 cm, the τ_d_ rapidly decreased to ~872, ~578, ~434 and ~352 hr, respectively (Fig. 3e). The variation of τ_d_ was plotted via the immersion depth (Fig. 3f). The graph shows an exponential decay relation between τ_d_ and the immersion depth. This tendency could be interpreted by effective pressure exerted on the air pocket(Supplementary Discussion 1)^46^,^47^. The exponential relations between hydrostatic pressure and effective pressure on the air pocket caused the exponential decay of τ_d_ by increment of immersion depth.

In case of SLIPS, the total reflection phenomena do not occur due to the absence of the air pockets. Therefore, the relative intensity method does not work for the underwater stability measurement of SLIPS.

### Drag reduction property of superhydrophobic surfaces

**Figure 4:**
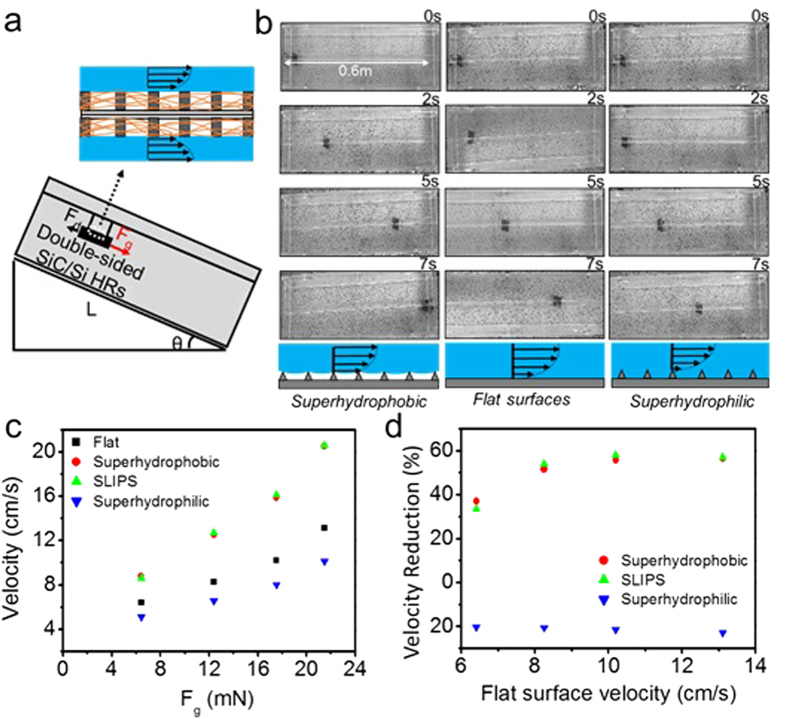
(**a**) Scheme of a set-up for drag reduction experiment. (**b**) Snapshots of movements of superhydrophilic and superhydrophobic SiC/Si hierarchical structures for 10 sec in drag reduction experiment, and scheme of a drag reduction mechanism for superhydrophobic surfaces. (**c**) Velocity variations depending on four different surface structures; flat, superhydrophobic, liquid infused slippery and superhydrophilic surfaces in various gravitational force (F_g_). (**d**) Velocity reduction ratio (Δ*V*) of three different surface structures; superhydrophobic, liquid infused slippery and superhydrophilic surfaces depending on the velocities of the flat surface.

To evaluate the drag reduction properties of the superhydrophobic SiC/Si hierarchical structures and their SLIPS samples, a measurement systemfor the sample velocity was designed, as illustrated in Fig. 4a (a detailed explanation isprovided in the method section). By allowing the double-sided superhydrophobic surfaces immersed in water to move along the tilted water filled tank, the velocity of the samples was measured and analyzed for drag reduction measurements. When the sink was tilted by θ°, the gravitational force (*F*_*g*_) exerted on the sample in the direction of sample’s movement is *F*_g_=*mg* sin *θ*, where *m* is massof the sample, and 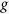 is the gravitational acceleration. When the substrate starts to move, a drag force caused by the contact between the solid surface and water layer is applied in the opposite direction of the movement. The drag force (*F*_*D*_) is expressed as


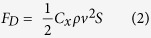


where *C*_*x*_ is drag coefficient of the substrate, *ρ* is the density of water, *v* is the velocity of the substrate and *S* is the area of the substrate. Thus the net force (*F*) applied on the sample could be


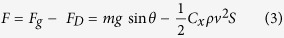


Figure 4b clearly shows direct observation of the effects of surface wettability on the sample velocity caused by drag reduction. Superhydrophobic SiC/Si hierarchical structures showed a higher velocity than that of flat Si surface and superhydrophilic SiC/Si hierarchical structures. At a 15° tilt angle, the superhydrophobic sample traveled 0.6 m in 7 sec, while the flat sample and superhydrophilic sample moved only 0.43 m and 0.35 m, respectively. This difference in the sample velocity results from the presence of the air interlayers on the superhydrophobic surfaces. The air interlayer hinders direct contact between the solid surface and water. This forms liquid/air/solid interfaces instead of liquid/solid interfaces, which causes a no-slip condition at the interface and drags the water flow on the surface (Fig. 4b).

The drag reducing effects of superhydrophobic surfaces, SLIPS and superhydrophilic surfaces were measured and compared at various velocities by altering the tilt angles of the sink to adjust the gravitational force. As the tilt angle increased from 15° to 60°, the velocities of the four type samples (flat Si surface, superhydrophobic SiC/Si surface, SLIPSon SiC/Si surface and superhydrophilic SiC/Si surface) also increased due to increase in gravitational force. The average velocities of each case after a travelling distance of 0.6 meters were plotted via *F*_g_ (Fig. 4c). In all different *F*_g_ s, the superhydrophobic surfaces and SLIPS showed the highest velocities while the superhydrophilic surfaces had the lowest ones. These results indicate that the superhydrophobic surfaces and SLIPS had drag reducing properties while superhydrophilic surfaces had drag enhancement properties. The decrease in the drag force on the superhydrophobic surfaces is due to the liquid/air/solid interface causing a slip condition as depicted in Fig. 4b. In case of SLIPS, the drag reduction results from the smooth and low surface energetic lubricant layers that prevent pinning and adhesion of the water layer. In case of superhydrophilic surfaces, the increased liquid/solid interfaces due to the high roughness and water affinity of the surfaces maximize drag force and minimize the velocities of the surfaces. To compare the drag reduction quantitatively, a velocity reduction ratio, Δ*V* was defined as 
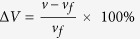
, where *v* is the velocity of the superhydrophobic surface or SLIPS or superhydrophilic surface and *v*_*f*_ is the velocity of the flat surfaces. The Δ*V* of three different surfaces (superhydrophobic surface, SLIPS and superhydrophilic surface) were calculated at tilt angles of 15°, 30°, 45° and 60°, respectively (Fig. 4d). With the superhydrophobic surfaces, the minimum Δ*V* value was 37% when flat surface velocity was 6.4 cm/s and increased gradually with the increment of the velocity. The maximum Δ*V* of the superhydrophobic surfaces was ~56% with a flat surface velocity of 13.1 cm/s. A similar behavior was observed for the SLIPS samples within the error range. The SLIPS samples had minimum Δ*V* of 33% with a flat surface velocity of 6.4 cm/s and a maximum value of 57% when flat surface velocity was 13.1 cm/s. On the other hand, the superhydrophilic surfaces showed steady decrease of Δ*V* from −20% to −23% as the velocity increased. It is noticeable that the velocity reduction ratio (in case of the superhydrophilic surfaces, velocity enhancing ratio) increased as the velocity of the substrate increased. The reason was that the effect of the drag force on the net force of the samples was amplified by the increase of the sample velocities because drag force was proportional to the square of the velocity (equation (2)).

### Regeneration of underwater superhydrophobicity and drag reducing property by photoelectrochemical reaction

**Figure 5:**
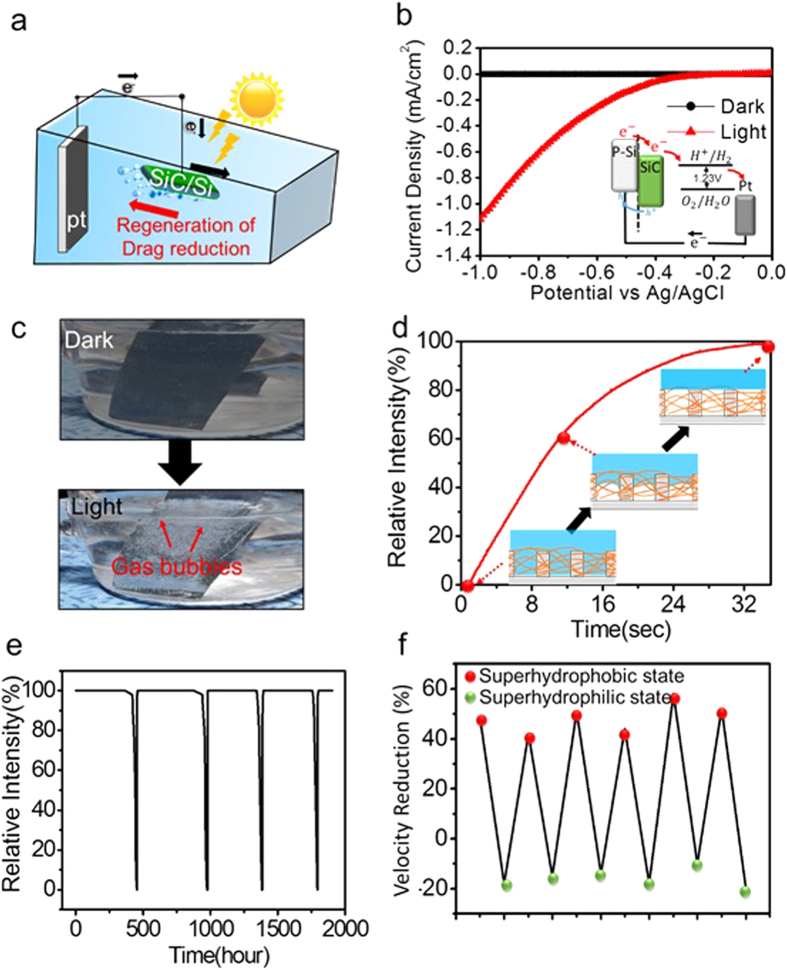
(**a**) A scheme of drag reduction regeneration system. (**b**) Photocurrent density vs applied potential in dark and light states. The inset image shows the schematic energy gap structures of SiC/Si hierarchical structures for the hydrogen generation process. (**c**) Digital images demonstrating the generation of hydrogen gas bubbles in the dark and light. (**d**) Relative intensity transitions of SiC/Si hierarchical structures during regeneration of underwater superhydrophobicity by photoelectrochemical reaction. The inset image is a scheme for wetting and dewetting processes of SiC/Si hierarchical structures. (**e,f**) Relative intensity and velocity reduction (Δ*V*) transitions of SiC/Si hierarchical structures for several wetting and dewetting cycles.

Although the superhydrophobic surfaces effectively reduce the drag force in water transport as mentioned above, they have limitations in real underwater applications due to the limited lifetime of the air interlayers. However, unlike SLIPS samples, superhydrophobic surfaces can be regenerated in underwater conditions. The lost underwater superhydrophobicity due to the collapse of the air interlayer could be restored by gas generation through photoelectrochemical (PEC) water splitting (Fig. 5a).The hydrogen gases generated by PEC reactions could be used to refill the lost air interlayer to restore superhydrophobicity. Figure 5b shows the photocurrent generation results for the PEC system with a SiC/Si hierarchical structure as a working electrode. The insulating SiO_2_ layers on the SiC/Si hierarchical structures were etched by HF treatment beforehand (Supplementary Fig. 4). All the PEC measurements were performed in a three-electrode PEC system with a Pt wire as a counter electrode and a saturated calomel electrode as a reference electrode under 1 Sun, AM 1.5 G illumination. The potential *vs* a reversible hydrogen electrode was analyzed using the Nernst equation and current density-potential (J-V) results were obtained. Because a p-type silicon substrate was used as the working electrode in our system, cathodic current generation occurred. Photogenerated electrons were used to reduce H^+^ ions to become H_2_ at the photocathode. Water was oxidized at the counter electrode. A schematic energy band diagram for our PEC system is displayed in the inset image of Fig. 5b. The digital images of Fig. 5c show the generation of gas bubbles in the dark and light. In the dark, the gas bubbles were not generated due to the absence of a light source. In contrast, exposure of light and high external bias (more than −2.0 V) on the samples resulted in the evolution of numerous gas bubbles on the surfaces.

The hydrogen gas generated by the PEC reaction on the SiC/Si hierarchical structures was used to refill the lost gas layers and restore underwater superhydrophobicity. As shown in Fig. 5d, the wetted SiC/Si hierarchical structures initially had a relative intensity of nearly 0%,but their relative intensity increased as the PEC reaction proceeded andwas eventually restored to ~100%. These results represented the successful regeneration of underwater superhydrophobicity of SiC/Si hierarchical structures with PEC. The generated hydrogen bubbles forced out the water that intruded in the structures and formed continuous gas layers on the entirety of the SiC/Si hierarchical structures (Inset images of Fig. 5d).

Figure 5e represents the changes in the relative intensity of the superhydrophobic SiC/Si hierarchical structuresover several wetting and dewetting cycles. The wetting process required hundreds of hours due to the high stability of the underwater superhydrophobicity, whereas regeneration occurred within a few seconds. The reproducible trend of relative intensity changes indicated that the underwater superhydrophobicity was completely restored by the PEC reaction over several cycles. Also the repeatable tendency of water CAs in the wetted PTFE-coated SiC/Si hierarchical structures before and after the PEC recovery reaction were measured and are shown in Supplementary Fig. 5.

The drag reduction property of SiC/Si hierarchical structures before and after the PEC reaction was also measured (Fig. 5f). The totally wetted SiC/Si hierarchical structures showed about a minus 20% drag reduction. After the regeneration process, the structures showed drag reduction of ~50% due to the regenerated gas layers. The drag reduction changes between the wetted and dewetted states of the SiC/Si hierarchical structures were repeatable and had the same tendency in several cycles. These resultsrepresent successful regeneration of drag reduction properties as well as underwater superhydrophobicity. Our study suggests that a successful drag reduction system can be developed by fabricating a smart regenerative superhydrophobic surface by applying a continuous PEC reaction.

## Conclusion

Regenerative drag reducing surfaces have been successfully developed by combining superhydrophobicity and solar water splitting. For comparison studies, we prepared three different samples (SiC nanowire arrays, Si micropost arrays and SiC/Si hierarchical structures) and their SLIPSs. Superhydrophobic SiC/Si hierarchical structures showed the highest stability of underwater superhydrophobicity due to their maximum Laplace pressure and interlocked net stack height. Next, the drag reducing effects of superhydrophobic surfaces and SLIPSs on SiC/Si hierarchical structures were measured. Due to the air interlayer of in the superhydrophobic surfaces and lubricant layer of SLIPS, both of these modifications enhanced drag reduction compared with that of flat surfaces. Conversely, the superhydrophilic SiC/Si hierarchical surfaces exhibited drag enhancement properties.Additionally, regeneration of the drag reducing property on superhydrophobic SiC/Si hierarchical samples was conducted using solar water splitting. The drag reduction efficiency was fully recovered with a PEC reaction of the SiC/Si hierarchical structures due to reformation of the air interlayer. This study presents a unique approach to overcome the critical problems of the limited stability of drag reduction and broadens the applicability of superhydrophobic surfaces in fluid transport systems.

## Methods

### Fabrication of SiC nanowire arrays

SiC nanowire arrays were grown on a p-type silicon wafer by carbothermal reduction of WO_3_ with graphite using NiO as a catalyst. The p-type silicon substrate was immersed in 0.01 M Ni(NO_3_)_2_/ethanol solution for 1 min and heated up to 60 °C for 30 min. This process repeated 3 times to obtain NiO catalyst deposited Si substrate. The NiO doped Si substrate, 2 g WO_3_ powders and 0.317 g graphite powders were put in the alumina boat placed in a two-zone furnace. The growth of SiC nanowire arrays was carried out at 1100 °C for 3 h under a 500 sccmAr flow.

### Fabrication of SiC/Si hierarchical structures

Common photolithography method was used for a growth of the Si micropost arrays with varying diameters of the post and distances between the posts. A negative photoresist (KMPR1050, Microchem) of 50 μm thickness was coated on a p-type silicon wafer by spin coating method. The substrate was baked to evaporate the coating solvent and densify the resist. A Cr photomask was placed on the substrate and photoetching was conducted with a uniform ultraviolet exposure illumination. The substrate was rinsed by acetone and trichloroethylene for removing remained photoresists. Si micropost arrays with varying diameter and pitches were patterned out of the photoresist. SiC nanowires were grown on these Si micropost arrays by carbothermal reduction method. The detailed method was same as above SiC nanowire fabrication section.

### Wettability control

Superhydrophobic SiC and SiC/Si substrates were obtained by the deposition of PTFE (polytetrafluoroethylene). Teflon AF solution (polytetrafluoroethylene (PTFE), Teflon Amorphous Fluoro-polymer 1600; copolymers of tetraflouroethlene, purchased from DuPont) was spin coated on the substrates at 2000 rpm for 30 s by 5 times. To obtain fully heat-cured PTFE, the substrates were heated up to 60 °C for 30 min. The PTFE treatment did not affect surface morphology and roughness of SiC nanowire arrays as shown in Supplementary Fig. 3. Liquid-infused surfaces were obtained by infusing fluorinated lubricant (GPL103, LOT-K2511, Dupont-Krtyox) in the structures. Several drops of the lubricant were cast onto the SiC and SiC/Si substrates, and dried at 60 °C for removing excess lubricant. The static water contact angle and sliding angle measurements were conducted to verify successful fabrication of superhydrophobic and liquid-infused SiC and SiC/Si surfaces.

### Underwater superhydrophobic stability analysis

The stability of underwater superhydrophobicity of SiC and SiC/Si surfaces was analyzed at varying immersion depth. The superhydrophobic surfaces were laid in the transparent water tank, tilted at 45°. The CCD camera was installed to record the images of submerged superhydrophobic surfaces during the analysis. The stability times of underwater superhydrophobicity of SiC and SiC/Si surfaces were measured by altering the sample immersion depth with 5, 10, 15 and 20 cm. The recorded images were converted to gray mode, and a threshold was applied. The threshold value was fixed at 225 for the entire images. The numbers of white and black pixels were measured, and the relative intensity was defined as the ratio of white pixels to entire pixels. Then the relative intensity data were plotted by time.

### Photoelectrochemical reaction

The photocurrent–voltage (I–V) measurements were performed in a three potentiostat system (potentiostat/galvanostat, model 263 A, EG&G Princeton Applied Research). A Pt mesh and a saturated calomel electrode was used as a counter electrode and a reference electrode, respectively. The as-prepared SiC/Si substrates were used as a working electrode and a 0.4 M Na_2_SO_4_ aqueous solution with nitrogen purged was used for electrolyte. The working electrode was illuminated with a solar simulated light source (AM 1.5 G filtered, 100 mWcm^−2^, 9ll60, Oriel) and the I–V curve was measured.

### Drag reduction analysis

To investigate drag reducing property of as-prepared surfaces, double-sided substrate were prepared. Two substrates with same surface property were pasted each other in opposite side to make the composite substrate have same surface property in both sides. The surface areas of all samples were fixed to 1.5 cm ×1.5 cm. The substrates were sunken in a sink fully-filled by water with a size of 0.6 m ×0.3 m ×0.2 m.A thin steel wire was used to connect the sample and the top of the sink to fix the immersion depth of the samples. One end of the wire was stuck on the sample and the other end of the wire was loosely hanged on the top of the sink. As the wire was loosely suspended on the top of the sink, the friction force was minimized. The sink was tilted in a degree of, 15°, 30°, 45°, and 60° respectively. Then the substrates were moving by the gravitation force. The substrates moved parallel to the tilted degree of the sink in a straight lane due to the rigid wire. The average velocities of the substrate were measured respectively. By comparing the velocities of flat, superhydrophilic, superhydrophobic and liquid infused substrates in each tilted degrees, the drag reducing effects were measured.

### Characterization

The morphology, structure, and composition of as-prepared structures were examined using field emission scanning electron microscopy (FESEM; JEOL, Model JSM 330 F), X-ray diffraction pattern (DMAX-1400 (Rigaku) at a fixed detector angle of 2°). The water contact angles and sliding angles were measured by 45 contact angle measurement system (Krűss, Model DSA-10) with 5 μl DI-water. The states of sample submerged underwater were recorded by a web-camera (@info, ALC-M1000).

## Additional Information

**How to cite this article**: Lee, B. J. *et al*. Bio-inspired dewettedsurfaces based on SiC/Si interlocked structures for enhanced-underwater stability and regenerative-drag reduction capability. *Sci. Rep.* **6**, 24653; doi: 10.1038/srep24653 (2016).

## Supplementary Material

Supplementary Information

## References

[b1] Banerjee I., Pangule R. C. & Kane R. S. Antifouling Coatings: Recent Developments in the Design of Surfaces That Prevent Fouling by Proteins, Bacteria, and Marine Organisms. Adv Mater 23, 690–718 (2011). 20886559 10.1002/adma.201001215

[b2] Xie Q. D. . Facile Creation of a Super-amphiphobic Coating Surface with Bionic Microstructure. Adv Mater 16, 302 (2004).

[b3] Kilian K. A. . Forming antifouling organic multilayers on porous silicon rugate filters towards in *Vivo*/*Ex vivo* biophotonic devices. Adv Funct Mater 17, 2884–2890 (2007).

[b4] Lafuma A. & Quere D. Superhydrophobic states. Nat Mater 2, 457–460 (2003). 12819775 10.1038/nmat924

[b5] Xu Q. F., Liu Y., Lin F. J., Mondal B. & Lyons A. M. Superhydrophobic TiO_2_-Polymer Nanocomposite Surface with UV-Induced Reversible Wettability and Self-Cleaning Properties. Acs Appl Mater Inter 5, 8915–8924 (2013). 10.1021/am401668y 23889192

[b6] Park H. K., Yoon S. W., Chung W. W., Min B. K. & Do Y. R. Fabrication and Characterization of Large-scale Multifunctional Transparent ITO Nanorod Films. J Mater Chem A 1, 5860–5867 (2013).

[b7] de Francisco R., Hoyos M., Garcia N. & Tiemblo P. Superhydrophobic and Highly Luminescent Polyfluorene/Silica Hybrid Coatings Deposited onto Glass and Cellulose-Based Substrates. Langmuir 31, 3718–3726 (2015). 25747277 10.1021/acs.langmuir.5b00293

[b8] Lee S., Kim W. & Yong K. Overcoming The Water Vulnerability of Electronic Devices: A Highly Water-Resistant ZnO Nanodevice With Multifunctionality. Adv Mater 23, 4398 (2011). 21960475 10.1002/adma.201101580

[b9] Lee S., Lee J., Park J., Choi Y. & Yong K. Resistive Switching WOx-Au Core-Shell Nanowires with Unexpected Nonwetting Stability Even when Submerged Under Water. Adv Mater 24, 2418–2423 (2012). 22488845 10.1002/adma.201200068

[b10] Sousa M. P. & Mano J. F. Superhydrophobic Paper in the Development of Disposable Labware and Lab-on-Paper Devices. Acs Appl Mater Inter 5, 3731–3737 (2013). 10.1021/am400343n 23581851

[b11] Joseph P. . Slippage of Water Past Superhydrophobic Carbon Nanotube Forests in Microchannels. Phys Rev Lett 97, 156104(2006). 17155344 10.1103/PhysRevLett.97.156104

[b12] Lee C. & Kim C. J. Maximizing the Giant Liquid Slip on Superhydrophobic Microstructures by Nanostructuring Their Sidewalls. Langmuir 25, 12812–12818 (2009). 19610627 10.1021/la901824d

[b13] Cao L. L., Jones A. K., Sikka V. K., Wu J. Z. & Gao D. Anti-Icing Superhydrophobic Coatings. Langmuir 25, 12444–12448 (2009). 19799464 10.1021/la902882b

[b14] Boreyko J. B. & Collier C. P. Delayed Frost Growth on Jumping-Drop Superhydrophobic Surfaces. Acs Nano 7, 1618–1627 (2013). 23286736 10.1021/nn3055048

[b15] Yin X. Y. . Integration of Self-Lubrication and Near-Infrared Photothermogenesis for Excellent Anti-Icing/Deicing Performance. Adv Funct Mater 25, 4237–4245 (2015).

[b16] Guo P. . Icephobic/Anti-Icing Properties of Micro/Nanostructured Surfaces. Adv Mater 24, 2642–2648 (2012). 22488894 10.1002/adma.201104412

[b17] Jung S., Tiwari M. K., Doan N. V. & Poulikakos D. Mechanism of Supercooled Droplet Freezing on Surfaces. Nat Commun 3, 615 (2012). 22233625 10.1038/ncomms1630

[b18] Zhang J. P. & Seeger S. Polyester Materials with Superwetting Silicone Nanofilaments for Oil/Water Separation and Selective Oil Absorption. Adv Funct Mater 21, 4699–4704 (2011).

[b19] Feng L. . A Super-hydrophobic and Super-oleophilic Coating Mesh Film for the Separation of Oil and Water. Angew Chem Int Edit 43, 2012–2014 (2004). 10.1002/anie.200353381 15065288

[b20] Zhang W. B. . Superhydrophobic and Superoleophilic PVDF Membranes for Effective Separation of Water-in-Oil Emulsions with High Flux. Adv Mater 25, 2071–2076 (2013). 23418068 10.1002/adma.201204520

[b21] Bhushan B. & Jung Y. C. Natural and Biomimetic Artificial Surfaces for Superhydrophobicity, Self-cleaning, Low adhesion, and Drag Reduction. Prog Mater Sci 56, 1–108 (2011).

[b22] McHale G., Newton M. I. & Shirtcliffe N. J. Immersed Superhydrophobic Surfaces: Gas Exchange, Slip and Drag Reduction Properties. Soft Matter 6, 714–719 (2010).

[b23] Truesdell R., Mammoli A., Vorobieff P., van Swol F. & Brinker C. J. Drag Reduction on a Patterned Superhydrophobic Surface. Phys Rev Lett 97, 044504 (2006). 16907578 10.1103/PhysRevLett.97.044504

[b24] Li X. M., Reinhoudt D. & Crego-Calama M. What do we need for a superhydrophobic surface? A review on the recent progress in the preparation of superhydrophobic surfaces. Chem Soc Rev 36, 1529–1529 (2007). 10.1039/b602486f 17619692

[b25] Feng L. . Super-hydrophobic surfaces: From Natural to Artificial. Adv Mater 14, 1857–1860 (2002).

[b26] Chen K. L., Zhou S. X., Yang S. & Wu L. M. Fabrication of All-Water-Based Self-Repairing Superhydrophobic Coatings Based on UV-Responsive Microcapsules. Adv Funct Mater 25, 1035–1041 (2015).

[b27] Carcouet C. C. M. C., Esteves A. C. C., Hendrix M. M. R. M., van Benthem R. A. T. M. & de With G. Fine-Tuning of Superhydrophobicity Based on Monolayers of Well-defined Raspberry Nanoparticles with Variable Dual-roughness Size and Ratio. Adv Funct Mater 24, 5745–5752 (2014).

[b28] Liu Y., Lin Z. Y., Lin W., Moon K. S. & Wong C. P. Reversible Superhydrophobic-Superhydrophilic Transition of ZnO Nanorod/Epoxy Composite Films. Acs Appl Mater Inter 4, 3959–3964 (2012). 10.1021/am300778d 22764733

[b29] Gao Y. Q. . Highly Transparent and UV-Resistant Superhydrophobic SiO_2_-Coated ZnO Nanorod Arrays. Acs Appl Mater Inter 6, 2219–2223 (2014). 10.1021/am405513k PMC3985694 24495100

[b30] Manna U., Carter M. C. D. & Lynn D. M. “Shrink-to-Fit” Superhydrophobicity: Thermally-Induced Microscale Wrinkling of Thin Hydrophobic Multilayers Fabricated on Flexible Shrink-Wrap Substrates. Adv Mater 25, 3085–3089 (2013). 23649745 10.1002/adma.201300341

[b31] Gauthier A., Symon S., Clanet C. & Quere D. Water Impacting on Superhydrophobic Macrotextures. Nat Commun 6, 8001 (2015). 26259509 10.1038/ncomms9001 PMC4918367

[b32] Lei W. W., Portehault D., Liu D., Qin S. & Chen Y. Porous Boron Nitride Nanosheets for Effective Water Cleaning. Nat Commun 4, 1777 (2013). 23653189 10.1038/ncomms2818

[b33] Rothstein J. P. Slip on Superhydrophobic Surfaces. Annu Rev Fluid Mech 42, 89–109 (2010).

[b34] Dong H. Y., Cheng M. J., Zhang Y. J., Wei H. & Shi F. Extraordinary Drag-reducing Effect of a Superhydrophobic Coating on a Macroscopic Model Ship at High Speed. J Mater Chem A 1, 5886–5891 (2013).

[b35] Choi K. S. . Turbulent Drag Reduction using Compliant Surfaces. P Roy Soc a-Math Phy 453, 2229–2240 (1997).

[b36] Bandyopadhyay P. R. . Experiments on the Effects of Aging on Compliant Coating Drag Reduction. Phys Fluids 17, 085104–085113 (2005).

[b37] Kim K., Adrian R. J., Balachandar S. & Sureshkumar R. Dynamics of Hairpin Vortices and Polymer-induced Turbulent Drag Reduction. Phys Rev Lett 100, 134504 (2008). 18517960 10.1103/PhysRevLett.100.134504

[b38] Dimitropoulos C. D., Dubief Y., Shaqfeh E. S. G., Moin P. & Lele S. K. Direct Numerical Simulation of Polymer-induced Drag Reduction in Turbulent Boundary Layer Flow. Phys Fluids 17, 011705–011708 (2005).

[b39] Thirumalai D. & Bhattacharjee J. K. Polymer-induced Drag Reduction in Turbulent Flows. Phys Rev E53, 546–551 (1996). 10.1103/physreve.53.546 9964284

[b40] Mavros P., Ricard A., Xuereb C. & Bertrand J. A Study of the Effect of Drag-reducing Surfactants on Flow Patterns in Stirred Vessels. Chem Eng Res Des 89, 94–106 (2011).

[b41] Hellsten M. Drag-reducing Surfactants. J Surfactants Deterg 5, 65–70 (2002).

[b42] Myska J., Stepanek P. & Zakin J. L. Micellar Size of Drag Reducing Cationic Surfactants. Colloid Polym Sci 275, 254–262 (1997).

[b43] Ferrante A. & Elghobashi S. On the Physical Mechanisms of Drag Reduction in a Spatially Developing Turbulent Boundary Layer Laden with Microbubbles. J Fluid Mech 503, 345–355 (2004).

[b44] Xu J., Maxey M. R. & Karniadakis G. E. Numerical Simulation of Turbulent Drag Reduction using Micro-bubbles. J Fluid Mech 468, 271–281 (2002).

[b45] Lee C. & Kim C. J. Underwater Restoration and Retention of Gases on Superhydrophobic Surfaces for Drag Reduction. Phys Rev Lett 106, 014502 (2011). 21231747 10.1103/PhysRevLett.106.014502

[b46] Poetes R., Holtzmann K., Franze K. & Steiner U. Metastable Underwater Superhydrophobicity. Phys Rev Lett 105, 166104 (2010). 21230986 10.1103/PhysRevLett.105.166104

[b47] Lee J. & Yong K. Surface Chemistry Controlled Superhydrophobic Stability of W_18_O_49_ Nanowire Arrays Submerged Underwater. J Mater Chem 22, 20250–20256 (2012).

[b48] Timonen J. V. I., Latikka M., Ikkala O. & Ras R. H. A. Free-decay and Resonant Methods for Investigating the Fundamental Limit of Superhydrophobicity. Nat Commun 4, 2398 (2013). 24025991 10.1038/ncomms3398

[b49] Bobji M. S., Kumar S. V., Asthana A. & Govardhan R. N. Underwater Sustainability of the “Cassie” State of Wetting. Langmuir 25, 12120–12126 (2009). 19821621 10.1021/la902679c

[b50] Marmur A. Underwater Superhydrophobicity: Theoretical Feasibility. Langmuir 22, 1400–1402 (2006). 16460052 10.1021/la052802j

[b51] Emami B., Tafreshi H. V., Gad-el-Hak M. & Tepper G. C. Effect of Fiber Orientation on Shape and Stability of Air-water Interface on Submerged Superhydrophobic Electrospun Thin Coatings. J Appl Phys 111, 064325–064331 (2012).

[b52] Xue Y. H., Chu S. G., Lv P. Y. & Duan H. L. Importance of Hierarchical Structures in Wetting Stability on Submersed Superhydrophobic Surfaces. Langmuir 28, 9440–9450 (2012). 22642584 10.1021/la300331e

[b53] Wang C. F., Tzeng F. S., Chen H. G. & Chang C. J. Ultraviolet-Durable Superhydrophobic Zinc Oxide-Coated Mesh Films for Surface and Underwater-Oil Capture and Transportation. Langmuir 28, 10015–10019 (2012). 22679902 10.1021/la301839a

[b54] Marmur A. Superhydrophobic and Superhygrophobic Surfaces: from Understanding Non-wettability to Design Considerations. Soft Matter 9, 7900–7904 (2013).

[b55] Senthil K. & Yong K. J. Enhanced Field Emission from Density-controlled SiC Nanowires. Mater Chem Phys 112, 88–93 (2008).

[b56] Lee J. & Yong K. Combining the Lotus Leaf Effect with Artificial Photosynthesis: Regeneration of Underwater Superhydrophobicity of Hierarchical ZnO/Si Surfaces by Solar Water Splitting. Npg Asia Mater 7, e201 (2015).

[b57] Walter M. G. . Solar Water Splitting Cells. Chem Rev 110, 6446–6473 (2010). 21062097 10.1021/cr1002326

[b58] Wolcott A., Smith W. A., Kuykendall T. R., Zhao Y. P. & Zhang J. Z. Photoelectrochemical Study of Nanostructured ZnO Thin Films for Hydrogen Generation from Water Splitting. Adv Funct Mater 19, 1849–1856 (2009).

[b59] Kwak G., Lee M., Senthil K. & Yong K. Wettability Control and Water Droplet Dynamics on SiC-SiO_2_ Core-Shell Nanowires. Langmuir 26, 12273–12277 (2010). 20509642 10.1021/la101234p

[b60] Wong T. S. . Bioinspired Self-repairing Slippery Surfaces with Pressure-stable Omniphobicity. Nature 477, 443–447 (2011). 21938066 10.1038/nature10447

[b61] Kamei J. & Yabu H. On-Demand Liquid Transportation Using Bioinspired Omniphobic Lubricated Surfaces Based on Self-Organized Honeycomb and Pincushion Films. Adv Funct Mater 25, 4195–4201 (2015).

[b62] Hao C. L. . Superhydrophobic-like Tunable Droplet Bouncing on Slippery Liquid Interfaces. Nat Commun 6, 7986 (2015). 26250403 10.1038/ncomms8986 PMC4918357

[b63] Vogel N., Belisle R. A., Hatton B., Wong T. S. & Aizenberg J. Transparency and Damage Tolerance of Patternable Omniphobic Lubricated Surfaces Based on Inverse Colloidal Monolayers. Nat Commun 4, 2176 (2013). 10.1038/ncomms3176 23900310

[b64] Rosenberg B. J., Buren T. V., Fu M. K. & Smits A. J. Turbulent Drag Reduction over Air- and Liquid- impregnated Surfaces. Phys. Fluids 28, 015103 (2016).

