# Supplementary material for: Bio-inspired dewetted surfaces based on SiC/Si interlocked structures for enhanced-underwater stability and regenerative-drag reduction capability
Source: Sci Rep. 2016 Apr 20;6:24653. doi: 10.1038/srep24653 (PMC4837397; doi:10.1038/srep24653)

Supporting Information

Bio-inspired dewetted surfaces based on SiC/Si interlocked structures for enhanced-underwater stability and regenerative-drag reduction capability

*By Junghan Lee, Zhuo Zhang, Seunghyun Baek, Sangkuk Kim, Donghyung Kim and Kijung Yong**

Surface Chemistry Laboratory of Electronic Materials, Department of Chemical Engineering, POSTECH (Pohang University of Science and Technology), Pohang, 790-784 (Korea)

* Corresponding author, Tel: +82-279-2278. Fax: +82-279-8169 E-mail: kyong@postech.ac.kr

Supplementary Figure 1: SEM images of Si micropost arrays

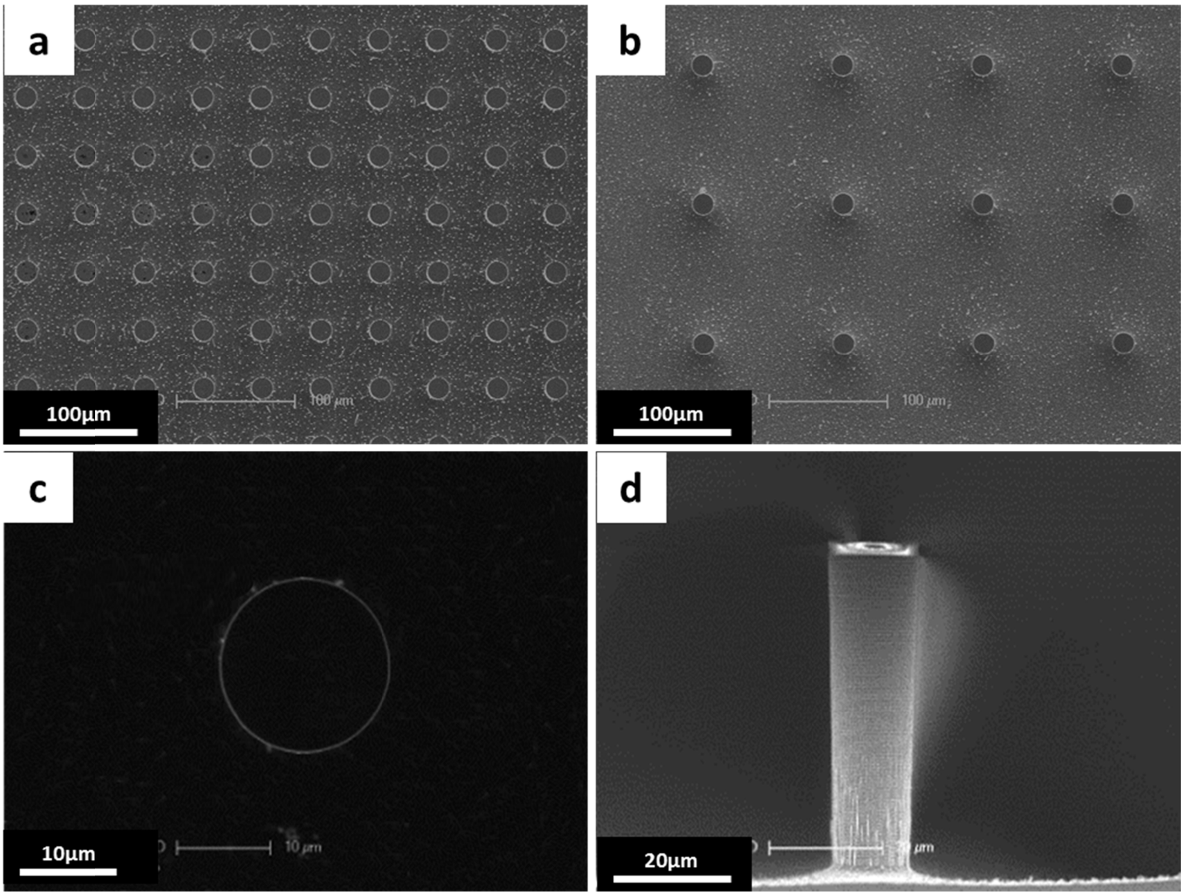

Supplementary Figure 1.SEM images ofa) Si micropost with 30μm pitches. b) Si micropost with 100μm pitches. c) magnified Si micropost. d) cross-sectional Si micropost.

Supplementary Figure 2: Fabrication of ZnO/Si hierarchical structures

ZnO nanowire arrays were synthesized using a simple hydrothermal method. A ZnO seed film with 50nm thickness was deposited on an FTO glass by radiofrequency (RF) magnetron sputtering using a ZnO target under 8.0×10^{3} torr Ar atmosphere at room temperature. The as-prepared substrate was immersed in an aqueous solution of 0.01M $ZnO(NO_3)_2 \bullet 6H_2O$ (98%, Sigma-Aldrich) and 0.2M NH_4OH (28 wt% NH_3 in water, 99.99%, Sigma-Aldrich) for 6 hours at 95℃. After the growth, the substrates were rinsed with deionized water and ethanol, then dried with N_2 gas.ZnO/Si hierarchical structures were grown on Si micropost arrays using same method described above.

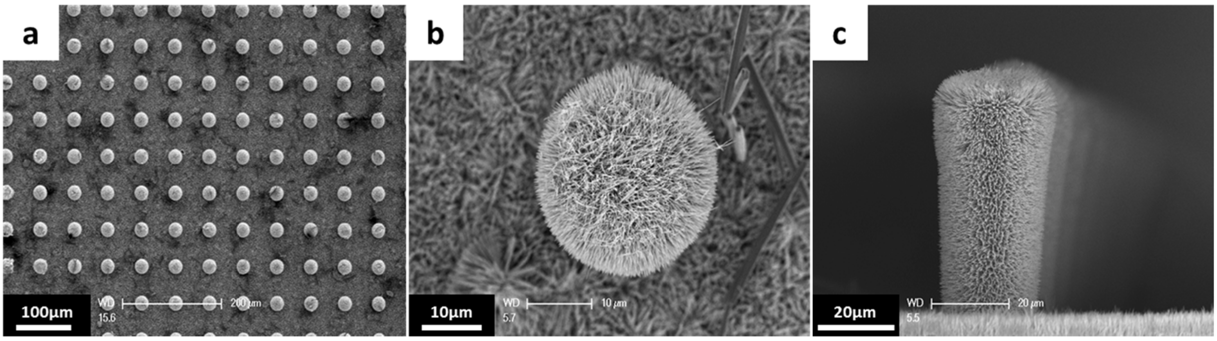

Supplementary Figure 2.a) top b) magnified c) cross-sectional SEM images of ZnO/Si hierarchical structures with micropost pitch spacings of 50 µm

Supplementary Figure 3: SEM images of SiC nanowire arrays after PTFE treatment.

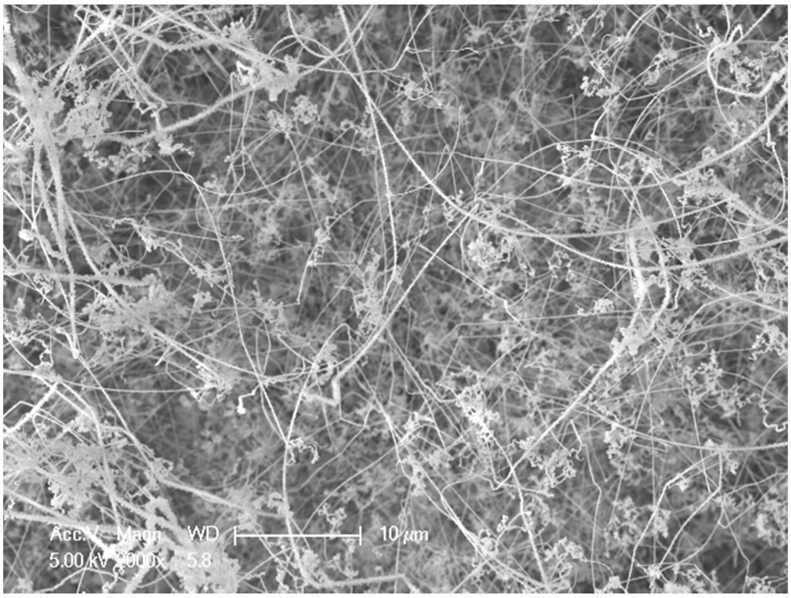

Supplementary Figure 3.SEM images of SiC nanowire arrays after PTFE treatment.

The PTFE treatment did not affect surface morphology and roughness of SiC nanowire arrays as shown in Supplementary Figure 3.

Supplementary Discussion 1: Relations between effective pressure and hydrostatic pressure.

The effective pressure that applied on the air pocket followed the equation,

$$\ln\left(\frac{p'}{p'_0}\right) = \frac{\nu}{RT}\left[p(h) - p_0\right]$$

where $p'$is the fugacity of the air, p'_0 is atmospheric fugacity, ν is the partial molar volume, R is the gas constant, T is the temperature, and p_0 is atmospheric pressure according to study of Poetes *et al*[43]. As the hydrostatic pressure is $p(h) = p_0 + \rho g h$ (where p_0 is atmospheric pressure, ρ is density of water, g is acceleration of gravity, h is immersion depth), the effective pressure exerted on the air pocket increased exponentially by the increase of immersion depth.

Supplementary Figure 4: HF treatment for removing SiO_2 layer on SiC/Si hierarchical structures.

As SiO_2 layer on SiC/Si hierarchical structures acted as insulating layer in photoelectrochemical reaction, SiO_2 layer was etched using a HF treatment. As prepared SiO2-SiC/Si hierarchical structures were immersed in a 9:1 H_2O:HF solution at room temperature for 5 min. The SiO_2 layer was fully removed through this process. It could be confirmed by comparing XRD data of SiC/Si hierarchical structures before and after HF treatment (Figure S3)

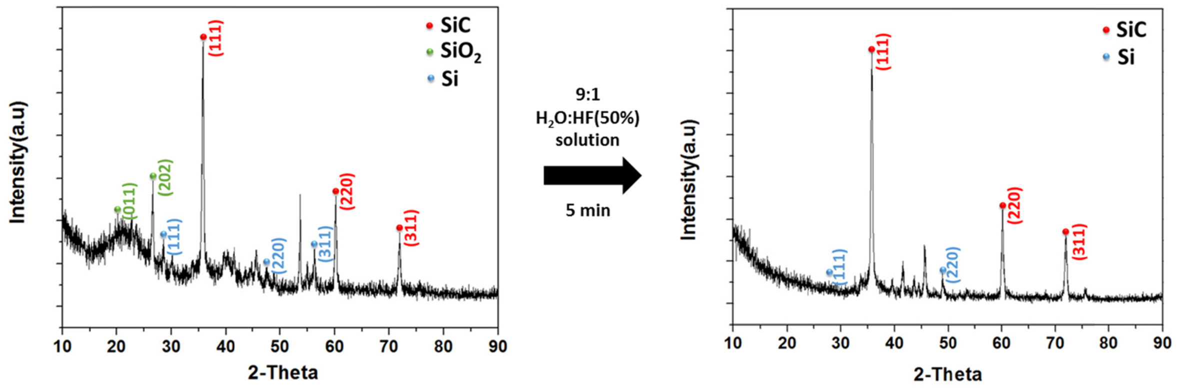

Supplementary Figure 4.XRD data of of SiC/Si hierarchical structures before and after HF treatment

Supplementary Figure 5: Water CAs transitions of SiC/Si hierarchical structures for several wetting and dewetting cycles.

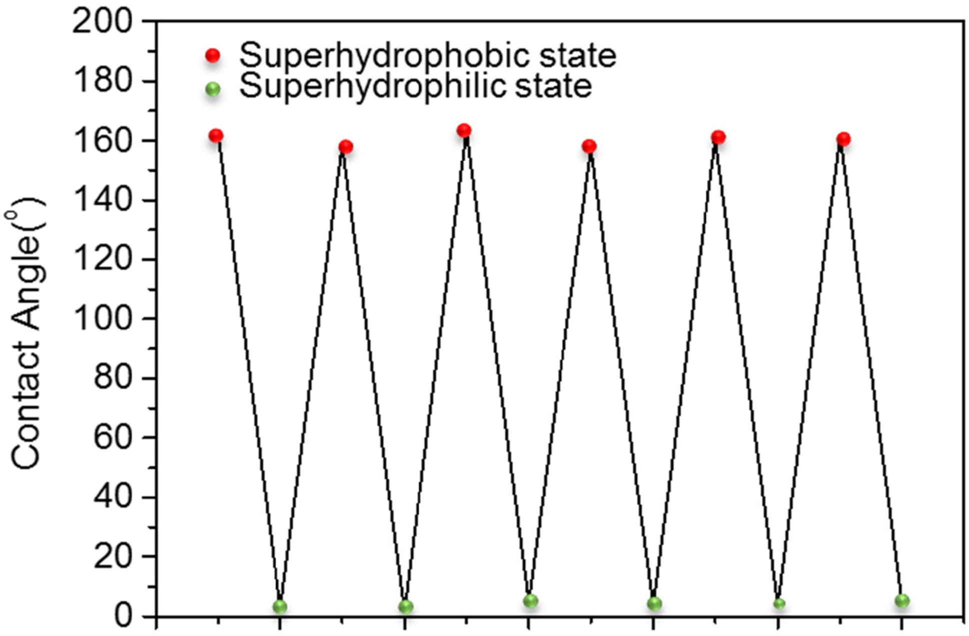

Supplement: Supplementary Information [file srep24653-s1.pdf]
